# Editorial: Executive functions, self-regulation and external-regulation: relations and new evidence

**DOI:** 10.3389/fpsyg.2023.1335354

**Published:** 2023-11-30

**Authors:** Jesús de la Fuente, Luis J. Fuentes, Flavia H. Santos, Maria Carmen Pichardo, Unai Díaz-Orueta

**Affiliations:** ^1^School of Education and Psychology, University of Navarra, Pamplona, Spain; ^2^School of Psychology, University of Murcia, Murcia, Spain; ^3^School of Psychology, University College of Dublin, Dublin, Ireland; ^4^School of Education, University of Granada, Granada, Spain; ^5^School of Psychology, Maynooth University, Maynooth, Ireland

**Keywords:** executive functions, self-regulation, self-regulated learning, external regulation, academic achievement

Recent research evidence has shown the importance of different psychological constructions for analyzing problems associated with lack of adequate behavior management in human beings. The model of the different levels of behavioral analysis – microanalysis level, molecular level and molar level – allows us to approach the study of executive functions, in relation to other constructions of said levels (de la Fuente et al., 2019).

The aim of this Research Topic was is to establish the necessary connections between the three levels of analysis with respect to the issue of human behavioral regulation. Many questions remain to be answered concerning the relationships and connections between the models mentioned: Does the construct of executive functions correspond linearly to that of self-regulation or self-regulated learning? How do they differ? What effect does the context have, when it is more or less regulatory? How do the types of relationships proposed by the molar theory relate to relationships identified at the molecular and microanalysis levels?

1) On the one hand, the *neuropsychological model* and its central variable, *executive functions* (EF) have become an essential construct for explaining learning difficulties and self-regulation of behavior in the lives of individuals. This well-documented construct represents the *level of microanalysis* of human behavior, which means that it focuses on the interaction between brain and behavior on cognitive performance, including decision-making across the lifespan. In parallel and complementary, other psychological models from research at the molecular and molar levels have been developed aiming to fill out the analysis and definition of behavior regulation, especially in the educational and health fields. A paper analized “*The executive function and effortful control, with similar and different evidence from big data analysis*” (Chae). Another article has analyzed the “Ice Cream,” a new virtual reality tool for the assessment of executive functions in children and adolescents, with a normative study (Fernandez et al.).

2) The behavioral models of *Self-Regulated Learning*, SRL, and general Self-Regulation modeled after the information processing paradigm have enabled us to accurately understand self-regulatory processes in the human being. These models, placed at a *molecular level of analysis*, give us a sequential, discrete understanding of self-regulatory behaviors, in the sphere of education and health. By analyzing across the behavioral sequence of before-during-after each act, the models have provided evidence of their value in assessment and intervention. Two works analyze the effect of Executive Functioning on school learning, both in Reading Comprehension (Leshem and Altman), and in English (Akhmedjanova and Moeyaert). Additionally, three articles have focused on the effect of self-regulated learning: the first, in asynchronous online learning situations (Sun et al.); the second report has analyzed the combined value of executive functions and self-regulated learning to predict differences in study success within higher education students (Manuhuwa et al.); a third work focused on noise reduction in preschool from a self- regulated learning perspective—implementation of a game-based voice regulation training program (Sarfaty and Ben-Eliyahu).

3) Finally, the behavioral model of *Self- vs. External Regulation, SR vs. ER Theory* takes its place at the *molar* level of analysis and has postulated the relevance of an interactive subject x environment analysis. This model has confirmed the relevance and value of the interaction of levels of regulation present in the subject and in their context, for predicting human behaviors in the fields of education and health. The continuum of Self-Regulation, Non-Regulation, Dys-Regulation (SR-NR-DR) has helped to operationally define the types of regulatory behavior, whether at the personal level or the contextual level. Two works focused on the effect of context on regulation have analyzed the factors affecting faculty conformity in South China universities (Xu and Chang) and the importance of teachers' supportive vs. undermining behavior for developments in early adolescents' self-regulation (Opdenakker). Additionally, two research reports have provided evidence regarding the combination of predictive factors of contextual and personal regulation regarding the variability of executive functions in university students (de la Fuente et al.) and the learning specific regulatory behavior (Pachón- Basallo et al.).

In conclusion, this Research Topic has provided a multilevel view of the executive functions construct, in relation to other related constructs, from a multilevel perspective. Future work should delve into this problem, since it is neither closed nor exhausted.

## Author contributions

JF: Conceptualization, Funding acquisition, Investigation, Project administration, Writing – original draft. LF: Conceptualization, Supervision, Writing – review & editing. FS: Resources, Supervision, Validation, Writing – review & editing. MP: Formal analysis, Methodology, Writing – original draft. UD-O: Conceptualization, Methodology, Validation, Writing – review & editing.

## References

[B1] de la Fuente J. González-Torres M. C. Aznárez-Sanado M. Martínez-Vicente J. M. Peralta-Sánchez F. J. Vera M. M. (2019). Implications of unconnected micro, molecular, and molar level research in psychology: the case of executive functions, self-regulation, and external regulation. Front. Psychol. 10, 1919. 10.3389/fpsyg.2019.01919 31507487 PMC6719524

